# A Case Study of Chronic Iliopsoas Tendinopathy and Sacroiliac Joint Dysfunction Masquerading As Pelvic Girdle Pain

**DOI:** 10.7759/cureus.15719

**Published:** 2021-06-17

**Authors:** Victoria M Mank, Javier Barranco-Trabi, Jeffrey K Mank, Jefferson Roberts, David P Newman

**Affiliations:** 1 Department of Internal Medicine, Tripler Army Medical Center, Honolulu, USA; 2 Department of Biology, University of New England, Biddeford, USA; 3 Department of Rheumatology, Tripler Army Medical Center, Honolulu, USA; 4 Pain Management-Physiotherapy, Interdisciplinary Pain Management Clinic, Tripler Army Medical Center, Honolulu, USA

**Keywords:** pelvic girdle pain, chronic pain, joint mobility, pelvic instability, pgp, lumbopelvic pain, symphysis pubis dysfunction, postpartum pain, lower back pain, pregnancy-related pelvic pain

## Abstract

Pain related to pregnancy can occur anytime between conception to the postpartum period. Pregnancy and the following months after birth are a time of physical change to the woman’s body, with significant hormonal effects. We present a case of a young female with chronic pain several years after her second pregnancy that presented a diagnostic challenge. She was initially diagnosed with persistent pelvic girdle pain (PGP) type 2, responded somewhat to appropriately targeted pelvic floor therapy, with a plateau in her progress. The diagnosis was revised to PGP type 4, with some improvement in pain with customized therapy. Her treatment again changed with a focus on sacroiliac joint (SIJ) dysfunction and iliopsoas tendinopathy with excellent and complete resolution of her pain. The overlapping nature of these diagnoses caused a significant challenge in creating a tailored physical therapy approach to her pain that eventually led to her final diagnosis being one of exclusion. Treatment was focused on optimization of joint mobility and tissue lengthening, with the resolution of her pain.

## Introduction

Pelvic pain related to pregnancy has a multitude of etiologies. Identifying the exact location of the pain can oftentimes be difficult for both the patient and the clinicians. Persistent pelvic girdle pain (PGP) following pregnancy is a relatively common condition that does not have a well-accepted etiology or treatment course [1]. PGP can begin to present anytime between the end of the first trimester and the first month post-delivery [2]. PGP covers a wide variety of diagnoses, including terminologies such as lumbopelvic pain, symphysis pubis dysfunction, pelvic instability, peripartum pelvic pain, pelvic instability, pregnancy-related PGP, and back pain of pregnancy [3]. Approximately 8-20% of women have persistent and debilitating pain two to three years following delivery, which falls under one of these diagnoses [3-5].

When diagnosing PGP, a cluster of symptoms can fall within this diagnosis, including generalized pain along the pelvic girdle, posterior pain localized to the sacroiliac joints (SIJs), pain along the pubic symphysis, or radiating pain down the inner thigh [6]. These locations, along with the chronicity of pain, can have a relatively long list of causes, with muscle and tendon strain from injury or exertion being deemed the culprits in many cases [7]. A chronic pain disorder has been linked to upwards of 80% of patients having an underlying musculoskeletal association [8]. Musculoskeletal pain can also be associated with underlying physical and mechanical changes, with a large differential, such as joint damage, osteoarthritis, and bone abnormalities, or due to a more broad or systemic pain syndrome such as fibromyalgia syndrome [8]. Diagnosing the underlying pathology involves obtaining a detailed history of the presenting discomfort, with a focus on the time course and any mechanism of injury, as well as physical examination. Descriptions of the pain can vary, but aching sensations or sharp pain is often described that can range from poorly localized to a specific location [9]. It is critical to rule out infectious, reactive forms of monoarticular arthritis, or targeted joint discomfort, which can be completed from appropriate past medical history collection, physical examination, and laboratory diagnostic testing [10]. Once the area of pain has been identified, be it within a joint, extra-articular, or referred musculoskeletal discomfort, and with appropriate history collection and physical examination, radiological imaging can be performed, which can be guided by the American College of Radiology Appropriateness Criteria [11]. Once physical examination, history, and time course with pregnancy have been identified, a differential may be narrowed to PGP or pregnancy-related low back pain. Differentiating these two will depend on character and severity of pain, location of pain, and physical examination pain provocation tests [3]. After performing pain provocation tests and diagnosing PGP, a classification system is in place describing five subtypes. Type 1 consists of pain along the anterior and posterior aspect of the pelvic girdle, pubic symphysis, and SIJs; type 2 consists of localized pain along the posterior pelvic girdle and bilateral SIJ; type 3 is pain consistent with posterior pelvic girdle and one SIJ; type 4 comprises pain along the pubic symphysis and anterior pelvic girdle; and type 5 which encapsulates inconsistent pelvic girdle findings [3,6].

We present a case of a female patient with a several-year history of chronic left hip pain thought to be related to a remote stress fracture, with worsening in her discomfort after pregnancy. This was attributed to PGP. She received appropriately targeted physical therapy based on her diagnosis. With her lack of improvement with treatment, the authors chose to adjust her treatment regimen towards a presumptive SIJ dysfunction with iliopsoas tendinopathy. A sequenced, multimodal intervention directed at the SIJ and iliopsoas tendon resulted in diagnostic clarity and the development of a successful impairment-based treatment program. The aim of this case report is to highlight the similarities in these diagnoses and provide treatment options that other providers may use in their clinical practice.

## Case presentation

A 23-year-old athletic female in the United States military presented with a five-year history of chronic left-sided anteromedial hip pain and urinary incontinence. The initial onset of pain was attributed to a grade II stress reaction of the proximal left femoral diaphysis at the adductor insertion from frequent running. While she did recover from this injury with rest and use of crutches, the patient had intermittent hip pain with running, hiking, and lifting. Over-the-counter non-steroidal anti-inflammatory medications, such as ibuprofen, did not improve the pain. The patient’s hip pain worsened one year prior to the presentation during her second pregnancy. Her symptoms worsened to include bilateral hip pain, low back pain, pelvic pain, and pelvic floor dysfunction. She completed a six-month course of pelvic floor physical therapy resulting in improvements in urinary continence, but not pain and function. A repeat left hip radiograph did not demonstrate any new fracture or stress injury. She had been seeing her primary care provider prior to her current presentation, who was able to determine that her current pain was unrelated to an infectious or rheumatological disorder. She was subsequently referred to an Interdisciplinary Pain Management Clinic (IPMC) for evaluation and management. The patient was evaluated by a pain management physician who recommended a comprehensive, multi-modal rehabilitation plan to include myofascial mobilization, consideration of platelet-rich plasma injections, yoga, and referral to the pain management physical therapist. 

Upon presentation to the clinic’s physical therapist, the patient’s goal was to run and exercise without pain. She wanted to explore all treatment options including another trial of physical therapy. The standard physical therapy evaluation and treatment practice incorporate structured history-taking to develop a working diagnosis and differential, a physical examination designed to rule in or out a specific diagnosis, and a rehabilitation program to address the impairments identified during the exam. Unfortunately, the patient’s pain level was elevated on initial presentation and the threshold for tissue irritability was low; therefore, the likelihood of the examination causing too much pain in which the patient would not want to engage in an exercise program was high. Instead, the examination was adjusted to maneuvers that were the least pain-provoking. 

Physical Examination

Physical evaluation at the IPMC revealed that the patient’s pain was localized to the left anteromedial hip at the inferior pubic ramus and the iliopsoas tendon as it passes under the ilioinguinal ligament to the insertion at the lesser trochanter. The patient’s average pain level was rated as a 4/10 on a visual analog pain scale. With this scale, the pain was measured with 0 indicating no pain and 10 indicating the worst pain the patient has ever experienced. She did not report any pain along the lumbar spine or SIJs or radiation of pain down her legs. A neurological clearing examination of L1-S1 revealed no dermatomal sensation changes or myotomal weakness in her bilateral lower extremities. A straight leg raise test was negative bilaterally. While standing, palpation revealed asymmetry in the patient’s pelvic landmarks with her posterior superior iliac spine (PSIS) and iliac crest elevated on the right side as compared to the left. The patient’s lumbar range of motion (ROM) was full and pain-free. Hip ROM was assessed passively with a goniometer and found to be within normal limits for all motions except extension. The hip extension was limited to five degrees on the left side. With moving of her leg passively into resistance, pain was reproduced. A positive flexion abduction external rotation (FABER) test was appreciated with decreased hip motion and pain. While standing, she was instructed to actively flex her hip towards her chest. As she lowered her leg, pain and a clicking sensation were appreciated with palpation over the iliopsoas tendon. During ambulation, she exhibited a Trendelenburg sign.

Motion Palpation Tests

The motion palpation tests utilized included the forward flexion test and Gillet’s test. In the forward flexion test, which was assessing for hypomobility of the SIJ, the test was positive when the PSIS on the involved side moved first when the patient bent forward. Gillet’s test, which was assessing for underlying sacroiliac dysfunction, was positive since the PSIS on the involved side did not move inferiorly as the patient flexed her hip actively.

Pain Provocation Tests

In the prone position, pressure was applied from a posterior to the anterior direction to the spinous processes from L1 to L5. No pain or segmental hypomobility was noted during the maneuver, indicating no pain referral from the lumbar spine. While supine, a thigh thrust test was performed to assess for SIJ dysfunction. With the patient’s knee and hip flexed, force was applied axially through the knee producing a shear force to the SIJ. The test was positive for pain and joint hypomobility. Finally, a piriformis test was performed to detect muscle tightness, which was found to be positive.

Strength Testing

Strength testing was performed while supine. Pain was partially reproduced with resisted hip adduction and flexion. In standing, the patient was instructed to push a 10-pound weight with her foot towards the right to simulate contraction of the hip adductors. This maneuver was selected as she reported an acute reproduction in her pain produced when she tried to move a 10-pound weighted box with her foot at work the previous week. She was unable to slide the dumbbell and her pain level increased to an 8/10 level, but quickly returned to her baseline of 4/10. This activity would serve later as a litmus test to determine the efficacy of selected treatment interventions directed at each impairment identified during the physical examination.

Diagnosis/Prognosis

The differential diagnosis specific to this patient’s symptoms upon initial evaluation to the IPMC included a hip adductor or flexor muscle strain or tendinopathy, intra-articular etiology (i.e., femoral acetabular impingement, osteoarthritis, or labral pathology), pelvic pain syndrome, snapping hip syndrome, or SIJ dysfunction. Given the lack of trauma at the time of her pain recurrence, a labral tear or other intra-articular pathology was excluded. Her pain reoccurred during her second pregnancy; therefore, SIJ dysfunction was considered high on the differential. However, the high level of pain localized to the anterior hip with pain reproduced with contraction of the hip flexor and adductors suggested a muscular pain generator, such as pregnancy-related PGP.

The prognosis for full resolution of symptoms was poor given the chronicity of symptoms, the poor response to previous conservative measures, and the short period of time that the patient would be available for care. The patient was planning to relocate in four weeks' time. 

Intervention

To validate the working diagnosis and identify contributing pain generators, an intervention-based diagnostic approach was utilized. First, biomechanical faults remote to the area of pain were addressed. Surrounding tissues to include the iliopsoas were treated to determine if this was the primary pain generator or contributing to her inguinal pain. After each intervention, the patient tried to move the dumbbell with her foot to validate if the tissue being assessed was a pain generator.

**Figure 1:**
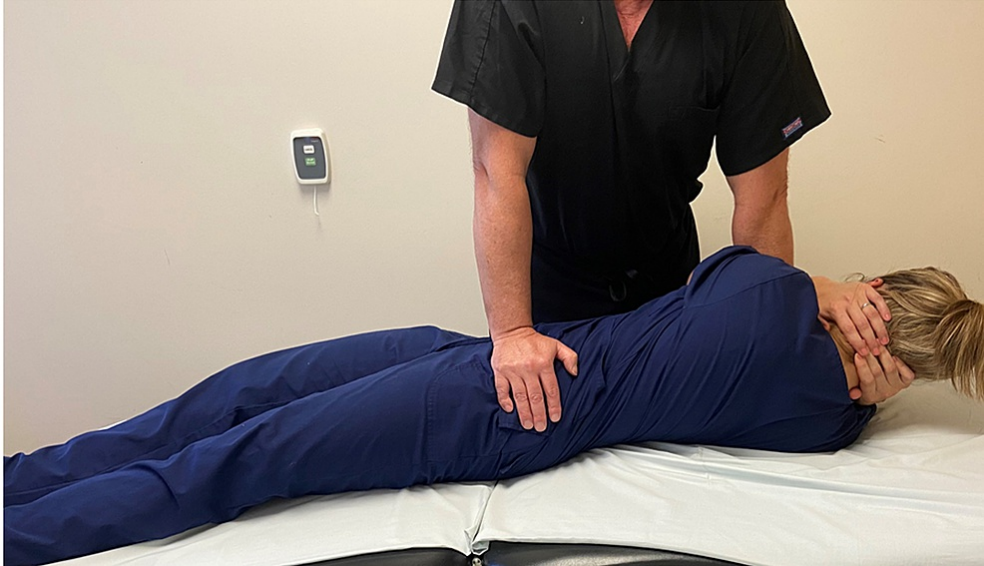
Sacroiliac joint manipulation technique. With the patient in supine, the provider passively side bends the patient to the left and then rotates the trunk to the right locking down the lumbar spine. The provider pushes the left ilium into a posterior direction until a barrier is felt. As the patient exhales, the provider imparts a high velocity, low amplitude force downwards. Photograph: Newman, DP. Sacroiliac Joint Manipulation Technique. Reproduced with permission of the author, 2021.

Upon initial treatment, the patient underwent a common osteopathic manipulation technique (OMT) directed at the left ilium to address the SIJ dysfunction (Figure 1). This technique allows the provider to apply a force at the ileum to posteriorly rotate it as the lumbar spine is locked down. The goal is to improve sacroiliac motion and improve a potential leg length discrepancy. After the maneuver, the patient reported less pain with contraction of the hip adductors against manual resistance, but was unable to move the dumbbell by contracting the left hip adductors in standing.

**Figure 2:**
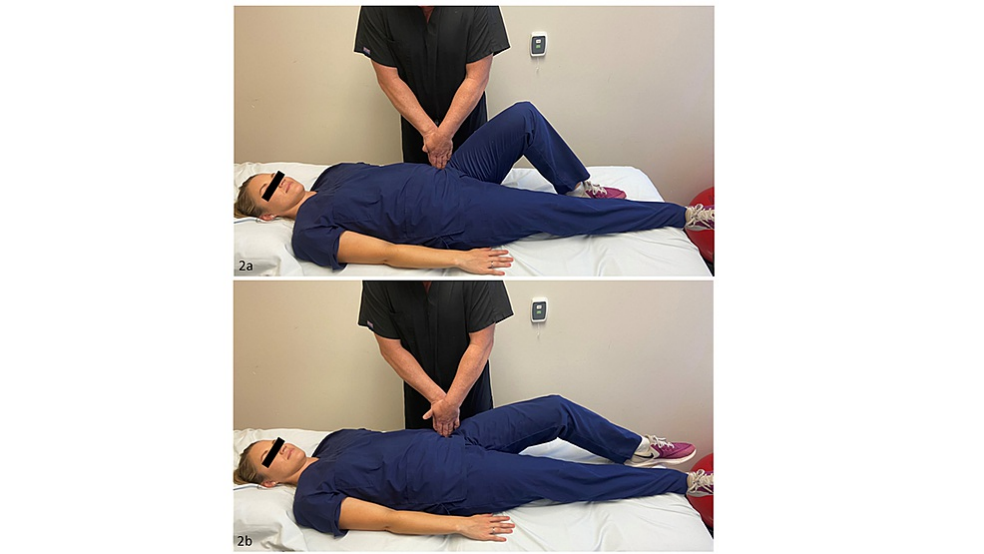
Iliopsoas active release technique. 2a: While the patient was supine with her hip flexed to 90 degrees, the provider applied digital pressure inferiorly and laterally pinning the iliacus muscle against the inside of the pelvic rim. 2b: The patient then actively extended her leg fully. The provider released the pressure and the patient returned her leg to the starting position. Photograph: Newman, DP. Iliopsoas Release Technique. Reproduced with permission of author, 2021.

To identify the impact of iliopsoas tightness upon the patient’s pain presentation and the effect of stretching upon achieving treatment success, an active release technique was applied to the iliacus muscle (Figure 2a and 2b). The active release technique artificially lengthens the muscle by decreasing tissue tension. By lengthening the tissue, the technique validates the importance of stretching the muscle in reducing pain. This procedure was repeated five times. The patient was then placed onto her right side, and the provider manually stretched her iliopsoas for five repetitions of 30 seconds. After this technique, the patient was able to move the dumbbell with a 1/10 pain level. 

The patient was started on a home exercise program consisting of piriformis stretching, iliopsoas stretching, and lumbopelvic stabilization exercises directed at improving muscle endurance of the abdominals, hamstrings, and hip adductors. The patient was asked to follow up one week later.

Due to the patient's work and scheduling conflicts, the patient was not able to follow up with physical therapy until two weeks later. Upon this visit, the patient reported the most pain relief that she had gotten over the last year. Her average pain level decreased by 50% to a 2/10. Pain with activity decreased from 8/10 to a 3/10 level. The iliopsoas stretching was reported to be the most effective exercise prescribed.

Physical examination revealed symmetrical pelvic landmarks and no joint hypomobility or pain derived during the thigh thrust test. Gillet’s and forward flexion tests were negative. Mild piriformis tightness was appreciated on the left side. The patient’s pain was mildly reproduced with passive hip extension with over pressure. The left iliopsoas was tighter than the right side. Active hip flexion in standing revealed a “click” consistent with snapping hip syndrome, but there was no pain. Contraction of the hip flexors and adductors in standing by moving the 10-pound dumbbell forward and medially with the foot did not reproduce her pain. The pain was reproduced with palpation to the iliopsoas tendon between the ilioinguinal ligament and lesser trochanter but not at the inferior pubic ramus.

**Figure 3:**
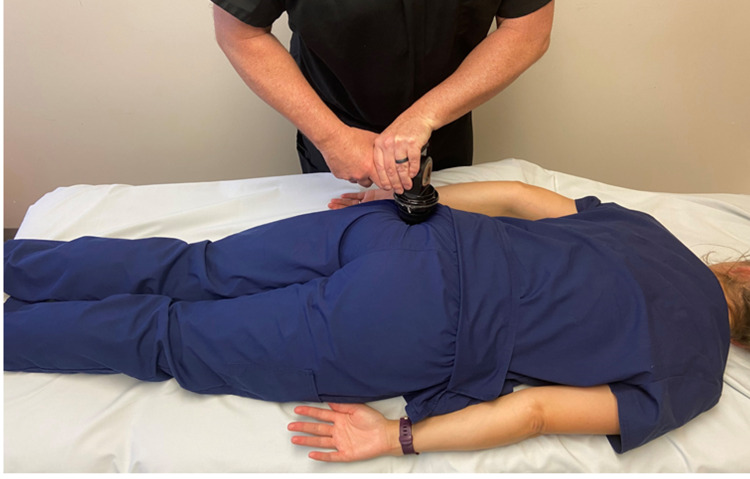
Instrument-assisted deep tissue mobilization to the piriformis muscle. While the patient was prone, the VibraCussor© was applied with downward pressure over the piriformis. The tool was moved superiorly and inferiorly along the course of the muscle just medial to the insertion at the greater trochanter to just lateral to the sacrum. Photograph: Newman, DP. Instrument-assisted deep tissue mobilization to the piriformis muscle. Reproduced with permission of author, 2021.

Treatment consisted of active release and manual stretching directed at the iliopsoas muscles for ten minutes. Right-sided piriformis tightness was addressed with instrument-assisted deep tissue mobilization using the VibraCussor® (IMPAC Inc, Salem, OR) directed at the piriformis for five minutes (Figure 3) followed by manual stretching for another five-minute period. The patient was instructed to continue her home exercise program and to go jogging prior to her follow-up in two weeks to determine her progress towards her functional goal.

Upon the third and final visit, the patient reported no pain at rest or with jogging. She ran three miles twice to validate that her goal had been met. The physical examination revealed iliopsoas tightness, but no pain was reproduced with resistance applied to the hip flexors or adductors. Pain was not reproduced with digital pressure over the iliopsoas tendon. She was encouraged to continue her stretching program at a minimum to optimize tissue length and reduce the snapping sensation. She was subsequently discharged from the IPMC.

## Discussion

Chronic pain is a broad category with multiple etiologies that requires careful evaluation of a patient, to determine if the pain is acute or chronic and to identify the exact location, triggers, and underlying pathology [12]. It is important to ask about the mechanism of injury and determine if the pain is more inflammatory or non-inflammatory. Narrowing it down to location, be it intra-articular or extra-articular, as was done in the case presented, assists with targeted imaging and therapeutic techniques. Clinicians must identify the time course and elicit any history of trauma or potential injury as to the cause of the discomfort. Once this is complete, appropriate laboratory testing and imaging must be chosen. Radiological imaging can often be beneficial if there are concerns for trauma or injury, but may not be necessary based on the history and physical examination. The American College of Radiology Appropriateness Criteria can be a useful tool when deciding which imaging to perform on a patient [11]. Laboratory testing can be used to target causes of pain to include infectious etiologies, inflammatory, or autoimmune pathologies. Our patient had completed her targeted testing and imaging prior to presenting to the IPMC and did not have signs or symptoms that would point towards an infectious, inflammatory, or autoimmune cause for her pain. In patients that are at higher risk, such as sexually active patients with a history of sexually transmitted diseases, it is reasonable to consider causes for the pain to include reactive arthritis or disseminated infectious etiologies. Other causes for a monoarticular include crystalline arthropathy or septic arthritis, especially in the correct population of patients with immunosuppression or underlying diabetes mellitus [10].

For the patient presented, her recurrent hip pain significantly worsened during her second pregnancy and included the low back and pelvic girdle. Pain localized to the pelvic girdle can have a wide spectrum of underlying pathologies, with hormonal and musculoskeletal changes of pregnancy altering the normal anatomy and physiology of a patient [6,13,14]. Low back and PGP can often go hand-in-hand, but the general consensus is that PGP is a subset of low back pain [15]. Our patient presented with multiple symptoms involving bilateral hips, low back, and pelvic pain. All of these terms have been used interchangeably, discovered through a literature review, to refer to PGP. The general instability of pregnancy, with hormonal changes involving ligament laxity and joint mobility, likely contributed to the patient’s presentation [15]. Theoretically, pregnancy causes the ligaments of the SIJ to slacken due to the hormone relaxin, which could further add to her chronic baseline pain [16]. However, current research does not have a consensus about specific hormones or serum levels that would cause or prevent PGP. It is thought that pregnant and postpartum females have joint and ligament hypermobility, which can add to their pain. Other risk factors for the development of PGP include parity, with each subsequent pregnancy increasing risk, metabolic factors such as diabetes mellitus, and genetic factors such as family members developing this condition appear to predispose patients for acquiring PGP [14]. After a careful review of the patient’s chart, physical examination, and history, her initial presentation and constellation of symptoms led to her original diagnosis of PGP, most closely resembling type 2, which involved bilateral SIJs and posterior PGP. Treatment for this would be focused on pelvic floor dysfunction, which she responded to appropriately with significant improvement in her urinary incontinence. However, she continued to have pain localizing now to her left SIJ and left iliopsoas tendon along the inguinal area. At this point, with her poor response to appropriately targeted treatment for PGP type 2, her treatment regimen and the diagnosis were changed in order to focus on PGP type 4 and likely SIJ dysfunction. Her pain now met the criteria for anterior pelvic girdle and pubic symphysis localization, i.e., type 4 PGP, and physical therapy treatments now focused on this area for manipulation and treatment. All exercises and manipulations were customized to the patient, with both her short timeline and improvement in function taken into account. Ultimately, her treatment was further adjusted with a focus on potential SIJ dysfunction after multiple physical examination maneuvers, including the thigh thrust test, localized her pain. With further customization of her treatment regimen, she was ultimately able to make a full recovery. This patient presented with a diagnostic dilemma, as her diagnosis and treatment plan changed many times. Initially, she was thought to have PGP type 2, was reclassified as type 4, and ultimately her treatment course was focused on SIJ dysfunction and iliopsoas tendinopathy. These diagnoses have significant overlap, as they all involve similar areas within the pelvic girdle, with many of the same signs and symptoms. She presented with a challenge, as she was able to respond to pelvic floor exercises that should have allowed relief from a diagnosis of PGP type 2. When she showed a plateau in her response, measured as continued pain, and her treatment was focused on a type 4 diagnosis, she had some improvement in pain, but still not complete resolution. The final diagnosis was SIJ dysfunction with iliopsoas tendinopathy, especially after pain was elicited during the thigh thrust test, and her focused treatment plan targeted the SIJ and iliopsoas tendon. SIJ pain has been described as referred pain from myofascial trigger points, especially when targeting areas around the gluteus, quadratus lumborum, rectus abdominis, piriformis, and iliopsoas muscles [17,18]. Our patient felt pain when performing iliopsoas-activating maneuvers and improvement following stretching exercises targeting this muscle. SIJ dysfunction has been shown to be associated with inflammatory and rheumatologic diseases, such as ankylosing spondylitis, psoriatic arthritis, or inflammatory bowel-associated seronegative spondyloarthropathies, but as in the case presented, micro-trauma can also be a link to chronic SIJ pain [17]. Micro-trauma has been described as caused by pregnancy and during the postpartum period, where the SIJ pain is attributed to the pubic symphysis being more unstable due to hormonal and physical body changes [17,19]. Micro-trauma can also be seen in athletic activity-related injuries and trauma [17]. This can be applied to the patient presented, as she had a history of a stress fracture in the general location of her pain several years prior to the current presentation. The build-up of the image-documented trauma in combination with her pregnancies and hormone-related changes to her SIJ, likely caused the instability and pain she reported. The referred pain to her iliopsoas muscle that improved following her exercise regimen supports the idea that she likely had SIJ dysfunction and tendon pathology.

Since PGP is a multifactorial process linked with hormonal changes, micro-trauma, age, genetic predisposition, body habitus, and underlying metabolic comorbidities and inflammatory pathologies, the most efficacious treatment should be a well-rounded, potentially multidisciplinary approach that is customized to the individual patient [15]. Undergoing a customized approach, it is appropriate to adjust treatment techniques for a potentially changing diagnosis. In our case, treatment changed from a focus on PGP to one of SIJ and iliopsoas tendinopathy. A functional approach to general joint stability in the location of the pain is critical to treatment in reducing laxity and stiffness [15]. This was achieved through targeted mobilization of the left SIJ and pelvic girdle stabilization exercise program that the patient would perform at home. Stabilization exercises focused on the pelvic girdle, which includes the SIJ, are critical in reducing pain. Tissue lengthening to address the iliopsoas pathology was achieved through active release techniques to inhibit the muscle tone and a course of stretching. These exercises have shown to be efficacious in reducing pain, as further evidenced by the patient presented [20].

The limitations of this study include the lack of external validity and the small sample size of only one patient being used for the techniques and exercises performed for relief of pain. There is also potential for selection bias due to the patient being lost to follow-up after treatment with no long-term assessment of her pain after she had moved away from the treatment facility. The conclusion of this case presentation is that the exercises, techniques, and physical manipulations used on the patient presented may be beneficial to others with PGP. Further studies with a larger sample size are needed to report definitive improvement in all patients with PGP.

## Conclusions

Hip and lower back pain are common problems, especially related to pregnancy. Chronic pain can be difficult to localize, and even more challenging to diagnose and treat. When considering a postpartum woman with chronic, poorly localized hip pain, a targeted approach can result in a diagnosis of exclusion. PGP, SIJ dysfunction, and iliopsoas tendinopathy were all considerations for the patient discussed in this case, with the final diagnosis and treatment course focusing on SIJ dysfunction and iliopsoas tendinopathy. With this diagnosis of exclusion, and trial and error involving her treatment plan, a multidisciplinary approach was required for her successful management. Pelvic floor dysfunction was relieved with the use of a pelvic floor physical therapist. Her subjective hip pain was finally relieved after a customized approach in her therapy with deep tissue mobilization, home and clinic-based iliopsoas stretching, and increases in her muscle endurance following her scheduled exercise program. A targeted, customized, and evolving approach to treatment is critical and may be beneficial for relieving pain in the poorly localized hip, back, and pelvic pain.
